# 

**Published:** 1888-02-04

**Authors:** 


CONTENTS.

**The Poor and their Doctors** - - - - - - 311

**Words of Consolation.** Chapter LXV.—The Sufferings of Christ - - - - - - - 312

**Talks with Nurses.** By M. M BASIL, M.A., M.B., Surgeon to the Southern Hospital, Manchester - - - - - 313

**A Working-Class View of Our Hospitals.** By the Chairman of Workmen's Governors, Sunderland Infirmary - 314

**The Doctor's Pulpit.**—Nursery Cares - - - - 315

**Scotch Physicians and Surgeons.** By C. G. FURLEY - 316

**National Pension Fund for Nurses** - - - 317

**Notes and News.**—The Hospitals Association.—The Jenny Lind Hospital.—Dr. H Burnes and Hospital Abuses—The Charité Hospital, Paris, and the Sœurs Augustines, etc. 318

**Everybody's Page** - - - - - - - 320

**Annotations.**—A Birching Machine.—Hindu Medicine.—Cottage Hospital Rules - - - - - - 321

**Amusements for Convalescents.**—Palmistry Notes. By a LADY - - - - - - - - 322

**Rachel Challice's Vow.** By LINA NEVILL, Author of "A Future on Trust," etc. Chapter VI.—A Wedding - - 323

**The Norwich Collection of Calculi** - - - 324

**The Nursing Mirror** - - - - - - 325

**Questions and Answers** - - - - - - 325

**Amusements with Profit for all Ages.**—For Men and Women.—Children's Corner.—Puzz es, etc. - - - 326

The average issue of "The Hospital" now exceeds **10,000 COPIES WEEKLY,** and the circulation is steadily increasing

